# Mindfulness improves inflammatory biomarker levels in older adults with mild cognitive impairment: a randomized controlled trial

**DOI:** 10.1038/s41398-020-0696-y

**Published:** 2020-01-21

**Authors:** Ted Kheng Siang Ng, Johnson Fam, Lei Feng, Irwin Kee-Mun Cheah, Crystal Tze-Ying Tan, Fadzillah Nur, Sin Tho Wee, Lee Gan Goh, Wei Ling Chow, Roger Chun-Man Ho, Ee Heok Kua, Anis Larbi, Rathi Mahendran

**Affiliations:** 1 Department of Psychological Medicine, National University of Singapore, Singapore, Singapore; 2 Department of Psychological Medicine, National University Hospital, Singapore, Singapore; 3 Department of Biochemistry, National University of Singapore, Singapore, Singapore; 4 Singapore Immunology Network, Agency for Science, Technology and Research, Singapore, Singapore; 5 Office of the President, National University of Singapore, Singapore, Singapore; 6 Department of Family Medicine, National University of Singapore, Singapore, Singapore; 7 Development Office, National University of Singapore, Singapore, Singapore

**Keywords:** Biomarkers, Neuroscience

## Abstract

Few randomized controlled trials investigated the effects of mindfulness intervention on older adults diagnosed with mild cognitive impairment (MCI). Furthermore, there have been hypotheses and theoretical mechanisms on the benefits of mindfulness intervention on biomarkers of stress, inflammation, and neuroplasticity implicated in MCI that warrant empirical evidence. We conducted a pilot randomized controlled trial to examine whether Mindful Awareness Practice (MAP) improved biomarker levels in older adults with MCI. Fifty-five community-dwelling older adults aged 60 and above were randomized into either the treatment arm, MAP, or the active control arm, the health education program (HEP). Researchers who were blinded to treatment allocation assessed the outcomes at baseline, 3-month, and 9-month follow-ups. Linear-mixed models were used to examine the effect of MAP on biomarker levels. MAP participants had significantly decreased high-sensitivity c-reactive protein (hs-CRP) levels at 9-month (*β* = −0.307, 95% CI = −0.559 to −0.054 *P* = 0.018). Exploratory sub-group analyses by sex showed significantly decreased hs-CRP in females only (*β* = −0.445, 95% CI = −0.700 to −0.189, *P* = 0.001), while stratification by MCI subtype showed hs-CRP decreased only in amnestic-MCI (aMCI) (*β* = −0.569, 95% CI = −1.000 to −0.133, *P* = 0.012). Although total sample analyses were not significant, males had significantly decreased interleukin (IL)−6 (*β* = −1.001, 95% CI = −1.761 to −0253, *P* = 0.011) and IL-1β (*β* = −0.607, 95% CI = −1.116 to −0.100, *P* = 0.021) levels at 3-month and non-significant improvements at 9-month time-point. MAP improved inflammatory biomarkers in sex- and MCI subtype-specific manners. These preliminary findings suggest the potential of mindfulness intervention as a self-directed and low-cost preventive intervention in improving pathophysiology implicated in MCI.

## Introduction

Mild cognitive impairment (MCI) is a transitional state between normal aging and very early dementia^1–3^. Owing to a rapidly aging population, the incidence of mild cognitive impairment (MCI) is expected to increase. Individuals with MCI have an increased risk of dementia, with 50% of MCI cases progress to develop AD^4^. Unfortunately, no new treatment options have been discovered in the past decade despite intensified efforts and numerous attempts in pharmaceutical trials^5^. Hence, the dementia field has recently moved towards validating potential preventative intervention to slow cognitive decline, before the irreversible symptoms of dementia and pathophysiology emerge. Early identification of MCI can prompt the prevention of dementia by improving the associated modifiable risk factors^6^. If the onset and progression of dementia could be delayed by just 1 year through any forms of interventions, there will be approximately 9.2 million lesser cases of dementia in 2050^7^. Additionally, novel intervention is imperative as MCI is an intermediate stage between being cognitively healthy and demented, thus representing a window of opportunity of which older adults may be still cognitively abled to acquire new techniques and a period of potentially malleable pathophysiology.

Mindfulness intervention as a preventative approach to improve psychiatric disorders and to delay dementia has gained traction in the past decade. A comprehensive meta-analysis of 209 studies concluded that mindfulness interventions with diverse participants afflicted by a range of psychiatric disorders are effective^8^, including depression, social anxiety, obsessive-compulsive, bipolar disorder, attention deficits disorder, and addiction^8–10^. Systematic reviews conducted by Gard et al.^11^ and Larouche et al.^12^ concluded that meditation interventions for older adults are feasible, with ample evidence, suggesting that meditation may potentially delay cognitive decline, thus delaying the progression of MCI and dementia. While one randomized controlled trial (RCT) reported trends of improvement in cognitive measures with MCI participants^13^, another more recent RCT showed significantly improved global cognitive scores in participants with MCI, upon completing a 1-year mindfulness intervention^14^. However, no studies have yet to examine the effect of mindfulness on peripheral biomarkers specifically in older adults with MCI, be it using blood or saliva samples. Furthermore, due to the inherent genetics and lifestyle differences, the effects of mindfulness intervention among Asian populations remained mostly unexplored^15^. Conversely, Kua et al.^16^ have demonstrated mindfulness practice was acceptable to Singaporean Chinese and did not carry the stigma of mental illness. In all, no mindfulness intervention focusing on cognition and peripheral biomarkers in older adults with MCI, utilizing parallel-group RCT design, has been conducted in Asian population.

Biologically, there have been various theoretical mechanisms on why mindfulness may be a favorable approach for MCI, which warrant empirical evidence. Several groups proposed^12,17,18^ that mindfulness may target inflammation, stress-related pathways, and neuroplasticity, thus reducing the risk of developing cerebrovascular disease and age-related neurodegeneration that could lead to the development of dementia. Indeed, MCI and dementia are likely to have multiple aetiologies, some of which are of cellular, metabolic, and endocrine origins^19^. Among them, systemic markers of inflammation are associated with cognitive decline in general and specific domains, both cross-sectionally^20^ and prospectively^21,22^. Since excessive neuroinflammation worsens during disease progression^19,23^, one of the biological pathways by which mindfulness intervention could delay the progression of MCI to dementia is through modulating inflammatory response^12,17,18^. Thus, future trials have been urged to examine inflammatory markers in MCI^23^. One of these potentially modifiable inflammatory factors associated with a heightened risk of and precede the onset of all-cause dementia is c-reactive protein (CRP)^24–31^. Despite its importance, only four mindfulness interventions targeted non-MCI populations have utilized CRP as a biomarker outcome measure in mindfulness intervention trials, with none of them showing a significant effect on CRP^10,32–34^. Two of these four studies^33,34^ showed statistical trends of *P* < 0.10, which warrant further investigations on the effects of mindfulness on CRP. Another closely related group of systemic pro-inflammatory biomarkers is the cytokines. Cytokines, particularly interleukin (IL)−6 and IL-1β, have been shown to be elevated in dementia patients and involved in the pathophysiologies of dementia^35,36^. Furthermore, mindfulness interventions ameliorated pro-inflammatory cytokines in various patient populations^37-40,39–43^. However, there is an apparent gap of knowledge on whether mindfulness could reduce the levels of pro-inflammatory cytokines in older adults with MCI specifically.

On neuroplasticity, peripheral brain-derived neurotrophic factor (BDNF) has been found to be significantly decreased in patients with Alzheimer’s disease (AD)^44,45^. On the other hand, higher BDNF level was associated with slower cognitive decline in both healthy older adults and patients with AD^46,47^. Several hypotheses have postulated the potential effects of mindfulness intervention on modulating neuroplasticity, through increasing BDNF levels^17,18,48,49^. Strikingly, none of the current randomized controlled trials (RCTs) have examined the effects of mindfulness intervention on BDNF levels in MCI participants^49^. Another closely related hypothesis, the allostatic overload model of the hypothalamic-pituitary-adrenal (HPA) axis, hypothesizes that chronic stressors initiate and accelerate the progression of cognitive impairment through the detrimental effects exerted by persistently elevated cortisol and decreased dehydroepiandrosterone sulfate (DHEA-S) levels^50,51^. Furthermore, salivary cortisol was also shown to be associated with worse cognitive performance^52^. However, there has been inconclusive evidence on the effects of mindfulness intervention on cortisol and DHEA-S levels in different target populations. No study insofar has examined the effect of mindfulness intervention on these biomarkers in MCI^12,18,34,53^.

To address these gaps of knowledge, we initiated an RCT of mindfulness intervention targeting older adults with MCI. Mindful Awareness Practice (MAP) is a Singaporean version of the mindfulness intervention^54^, modeled on the didactics of McBee^55^. One of the two aims of MAP-RCT was to examine the effects of Mindfulness Awareness Practice (MAP, the treatment arm) in improving biomarkers in older adults with MCI, in comparison to the Health Education Program (HEP, the active control arm). We hypothesized that MAP could: 1) decrease CRP, IL-1β, IL-6, and cortisol levels and (2) increase BDNF and DHEA-S levels in community-dwelling older adults with MCI.

## Methods

### Study sample, screening, and recruitment

This study was approved by the National University of Singapore ethics committee, Institutional Review Board (NUS-IRB Reference No: B-14-110), and registered with the clinical trial database (https://clinicaltrials.gov/ct2/show/NCT02286791). The participants were older adults aged 60 and above, who have participated in the longitudinal follow-up study, Diet and Healthy Ageing Study (NUS-IRB Reference No:10–517), at the Training and Research Academy at Jurong Point (TaRA@JP), a community-based research center established by NUS Psychological Medicine department. The research nurses, research assistants, and a Ph.D. student obtained informed consent before screening for potentially eligible participants. The screenings for eligibility were performed based on a priori inclusion and exclusion criteria. Eligible participants were then randomized by an independent research assistant that was not affiliated with the trial using the Random Allocation Software version 2.0 (Saghaei, Isfahan, Iran) to randomly allocate the participants in 1:1 ratio to either the mindful awareness practice (MAP) or health education program (HEP) arm, using a random number generator. The study’s research co-ordinator assigned the participants to the interventions. A single-blind design was employed; The assessors were blinded to the study arm assignments while the participants were aware of the study arms they were assigned to.

### Inclusion and exclusion criteria

The inclusion criterion was fulfilling the operational criteria of MCI based on The Diagnostic and Statistical Manual of Mental Disorders, Fifth Edition (DSM-V)^56^. We excluded older adults with either dementia or normal aging, had a neurological or major psychiatric condition, had a terminal illness, had visual or hearing impairments, had upper and lower limb motor difficulties, and those who were participating in another intervention at the time of the screening. To derive the cognitive status of the participants, there was a two-tier procedure. First, the assessors, comprises a team of trained research assistants and a Ph.D. candidate, administered the clinical dementia rating (CDR) and neurocognitive assessments (NCA) to all screened participants at the research center and derive at preliminary research diagnosis. Subsequently, final research diagnoses of MCI were made during the study’s consensus meetings by a panel consisting of at least two consultant-ranked psychiatrists, clinical scientists and the trained assessors who administered the tests. CDR-Sum of Box (CDR-SoB) was calculated as it has been demonstrated to effectively and accurately stage MCI and dementia severity^57^.

### Intervention

We employed a parallel arm RCT as the study design. For the first 3 months, the sessions were more frequent and were held weekly over 12 weeks, with each session of the MAP and HEP arms spanning approximately 1 h. From 3-month to 9-month, six monthly booster sessions were held for both arms. Attendance was recorded. Additionally, the participants were provided with personal diaries to record their practices at home and were asked to return them at the subsequent sessions to measure the adherence to daily practice and frequencies of home practice.

### Treatment arm: Mindful Awareness Practice (MAP)

Mindful Awareness Practice techniques were modeled on the didactics of McBee’s mindfulness-based elder care (MBEC)^55^, which adapted the techniques to the unique needs of the older adult population. Different from MBEC, mindfulness-based stress reduction (MBSR) is targeted at the general population and is not restricted to older adults. It also assumes that the participants are able to understand and follow instructions, have a good attention span, are able to commit to the experience and to participate in some form of exercise. Older adults often are not able to fulfill the above criteria^58^. MBEC made some adaptations to the MBSR model while maintaining the core intention of mindfulness^58^.

During each MAP session, participants were guided by a certified instructor to engage in these mindfulness techniques and were requested to practice the techniques at home daily. In MAP, we employed various mindfulness techniques, among them mindfulness of the senses practice, mindful breathing, and body scan practice, movement nature meant practice, visual-motor coordination tasks, and mindful stretching.

### Control arm: Health Education Program (HEP)

Similar to the HEP proposed by MacCoon et al.^59^, HEP encompassed topics pertinent to the general health of older adults, which included sleep, diabetes, hypertension, healthy diet, medications, depression, complications of diabetes and hypertension, anxiety, exercising, coping with grief and stress, social support and connectedness, and dementia. The program was delivered by a panel of healthcare professionals specialized in the topics, which included clinicians, nurses, and psychologists. The use of health education as the active control arm was recommended to control for non-intervention-specific components^60^.

### Outcome measurements

Bio-specimens were collected at baseline, 3-month, and 9-month, corresponded to the start of the trial, the end of the weekly intervention and the end of the monthly intervention, respectively. The primary outcome measurements were six biomarkers.

### Bio-specimen collections

Two types of bio-specimens, blood and saliva were collected. Blood and saliva collections were scheduled between 9:00 and 11:00 a.m. in the morning to minimize diurnal variations^34,61^. For fasting blood, the participants stopped the consumption of foods after 10 p.m. the night before venepuncture. The consumption of only water was advised. The participants were advised not to exercise or perform rigorous physical activities before the collections and not to rush to the center in the case that they were late. Blood draw via venepuncture was performed by the research nurses on the day that the participants visited the research center. The blood was kept at 4 °C for a maximum of three hours before being processed in the laboratory. Unstimulated and whole saliva samples were collected by the research nurses on the same day of venepuncture, to maintain the consistency and quality of the saliva samples. Passive drool collection procedures were employed^62^; The participants were instructed to pool and accumulate the saliva in the floor of the mouth, before passively drooling the saliva into a Falcon™ 15 ml Conical Centrifuge Tube (Fisher Scientific, USA). Immediately after the collection, the saliva samples were frozen at –20 °C until being further processed. We controlled for a number of pre-analytical variables systematically by having a pre-analytics saliva collection protocol, including the following instructions given to the participants: no consumption of a major meal within 60 min prior to collection, only drinking of plain water was advised and rinsing of mouth with plain water to remove food residues 10 min before collection. Any contamination with blood was also visually inspected^63^ after the sample collection and before the samples were processed.

### Biomarker pre-processing, storage, and measurements

The blood samples were sent to the laboratory located at Singapore Immunology Network (SIgN). Subsequently, the whole blood samples were centrifuged at 1650 × *g* for 25 min at room temperature to obtain the plasma. The plasma samples were then stored at –80^ o^C until further analyses. Saliva processing followed similar procedures, according to the manufacturer’s instruction (Salimetrics, Pennsylvania, USA). The frozen saliva samples, which were stored at –20^ o^C upon sample collection, were transported in batches to the laboratory for sample processing. Upon reaching the laboratory, the saliva samples were thawed on ice and were subsequently centrifuged at 3000 × *g* for 15 min. The supernatants containing clear saliva were then aliquoted and stored at –80 ^o^C until further analyses (Salimetrics, Pennsylvania, USA). After sample collections from all the three time-points were completed, all samples for the same participants from different time-points were assayed on the same day and on the same plates, to avoid batch effects. Biomarkers for this trial were examined using commercially available enzyme-linked immunosorbent assay (ELISA) kits. A total of six biomarkers were measured. The three blood-based biomarkers measured were high-sensitivity (hs)-CRP (Tecan, Männedorf, Switzerland), BDNF (Promega Corporation, Madison, USA) and DHEA-S (CUSABIO, Houston, USA). Salivary cortisol, IL-1β, and IL-6 were assayed using validated ELISA kits for measuring salivary biomarker levels (Salimetrics, Pennsylvania, USA). All the experiments were performed as per the instructions of respective manufacturers of the kits.

### Statistical analyses

Based on previous studies^33,34,41^ examining the effects of mindfulness on the biomarkers chosen for this study, we postulated the effect size on the selected biomarkers to be 0.5. Hence, we required 24 participants for each arm to have a power of 80% to detect statistical significance at 5% level. Considering potentially 20% drop-out rate, 30 participants needed to be assigned to each arm at baseline. Hence, the targeted total sample size was 60. The biomarker levels were expressed as mean ± standard error (SE). The differences in baseline variables were examined using Student’s *t*-test, chi-square or Fisher’s exact tests according to the nature of the data. The raw values of the biomarker measurements did not fulfill the normality assumption; therefore, all the raw values of the biomarkers were natural log-transformed for subsequent analyses and were successfully normalized, based on dot plots, skewness, and kurtosis. Linear-mixed model was employed to examine the treatment effects of MAP. In each of the models, the outcome of interest was entered as the dependent variable. Baseline values of the respective outcome variable, age, sex, years of formal education, time-points of the intervention, treatment arm, and time-points and treatment arm interaction term were included as covariates for all the models. Additional covariates relevant to MCI and dementia, including cardiovascular diseases, history of myocardial infarction, geriatric depression scale (GDS), and geriatric anxiety inventory (GAI) clinical cutoffs, were added to examine their effects on the baseline models, based on model fits using the Akaike information criterion (AIC) and Bayesian information criterion (BIC) values. In the final models, for all the additional covariates, only the significant ones were retained. We further performed exploratory sub-group analyses, by stratifying the whole sample separately by sex and MCI subtypes, namely amnestic and non-amnestic MCI (aMCI and naMCI), to explore their potential modifying effects on the outcomes. The participants did not have to complete all the sessions to be included in the analysis, and the attendance rate was tested as a covariate. All the analyses were based on intention-to-treat principle, performed using the Statistical Package for the Social Sciences (SPSS) Statistics for Windows, version 23.0 (IBM Corp., Armonk, N.Y., USA). For all the analyses, a two-tailed *P*-value of < 0.05 was considered statistically significant. Owing to the pilot and exploratory nature of this study to examine the biomarkers potentially modifiable by MAP, we did not control for multiple testings^64^. Other pilot RCTs of exploratory and hypothesis-generating nature have adopted similar practice^65,66^.

## Results

### Baseline demographics and characteristics

**Fig. 1:**
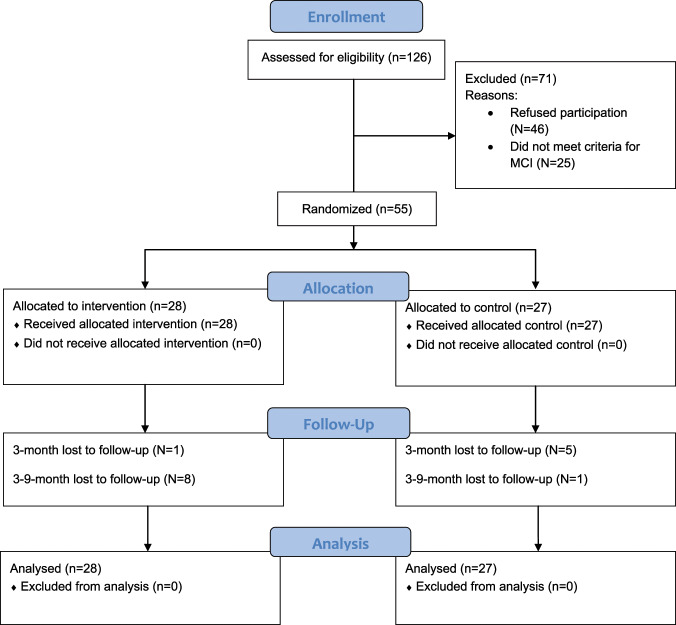
CONSORT flow diagram for MAP-RCT.

The study flow was illustrated in a CONSORT Flow diagram (Fig. 1). We recruited a total of 55 participants aged 60 to 86 (mean = 71.28 years, SD = 6.00). No significant differences in all baseline variables were observed, which included age, sex, and education levels (Table 1).

**Table 1:** Comparisons of the baseline demographic and other characteristics between participants in the Mindful Awareness Practice (MAP) and Health Education Program (HEP) Arms (*N* = 55).

Baseline demographics and characteristics	MAP, treatment (*N* = 28)	HEP, control (*N* = 27)	*P*-value
Age, mean (SE)	71.89 (1.14)	70.67 (1.19)	0.46
Sex, *N* (%)			
Male	8 (28.60%)	6 (22.20%)	0.59
Female	20 (71.40%)	21 (77.80%)	
Education, *N* (%)			
No formal education	15 (55.60%)	20 (74.10%)	0.33
Primary school	6 (22.20%)	3 (11.10%)	
Secondary school/ITE	3 (11.10%)	4 (14.80%)	
Junior college / polytechnic	1 (3.70%)	0 (0%)	
University and postgraduate	2 (7.40%)	0 (0%)	
BP (systolic), mmHg, mean (SE)	135.50 (4.60)	141.17 (3.46)	0.33
BP (diastolic), mmHg, mean (SE)	71.33 (2.11)	72.61 (1.87)	0.65
Pulse rate, BPM, mean (SE)	72.37 (1.99)	69.48 (1.85)	0.29
BMI, kg/m^2^, mean (SE)	24.76 (.85)	24.06 (.67)	0.53
Ethnicity, *N* (%)			
Chinese	27 (96.40%)	27 (100%)	1.00
Indian	1 (3.60%)	0 (0%)	
Others	0 (0%)	0 (0%)	
Employment status, *N* (%)			
Retired	14 (51.90%)	11 (40.70%)	0.14
Full-time worker	0 (0%)	0 (0%)	
Part-time worker	0 (0%)	4 (14.80%)	
Housewife	13 (48.10%)	12 (44.40%)	
Marital status, *N* (%)			
Single	1 (3.70%)	0 (0%)	0.29
Married	18 (66.70%)	14 (51.90%)	
Divorced	2 (7.40%)	1 (3.70%)	
Widowed	6 (22.20%)	12 (44.40%)	
CDR-sum of box, mean (SE)	0.61 (0.06)	0.44 (0.06)	0.05
MMSE (total scores), mean (SE)	24.59 (0.63)	24.70 (0.75)	0.91
GDS, *N* (%)			
<5	18 (64.30%)	22 (81.50%)	0.15
≥5	10 (35.70%)	5 (18.50%)	
GAI, *N* (%)			
<9	21 (77.80%)	26 (96.30%)	0.10
≥9	6 (22.20%)	1 (3.70%)	
Attendance rate (%)	88.6 (12.48)	87.0 (19.11)	0.77
MCI subtypes			
aMCI	13 (46.40%)	8 (29.6%)	0.27
naMCI	15 (53.6%)	19 (70.4%)	
Total number of metabolic disorders	1.44 (0.22)	1.52 (0.16)	0.79
Presence of diabetes	6 (22.2%)	8 (29.6%)	0.76
Total number of chronic diseases	2.04 (0.33)	2.85 (0.25)	0.66
Total number of medications taken	2.89 (0.44)	2.96 (0.39)	0.90
Total number of participants taking psychotropic medications	1 (3.70%)	0 (0%)	1.00

*BP* blood pressure, *BPM* beats per minute, *BMI* body mass index, *MMSE* mini-mental state examination, *GDS* geriatric depression scale, *GAI* geriatric anxiety scale, Clinical cutoffs for GDS and GAI are 5 and 9, respectively, *aMCI* amnestic MCI, *naMCI* non-amnestic MCI.

### Effects of MAP Intervention on Biomarker Levels

**Fig. 2:**
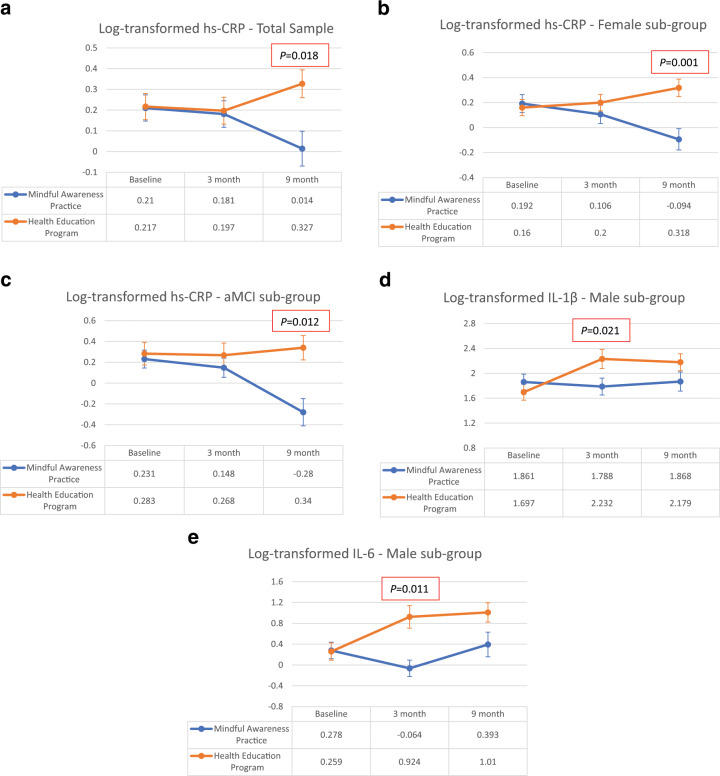
Changes in log-transformed biomarker levels across baseline, 3-month, and 9-month time-points in the Mindful Awareness Practice (MAP) arm, compared to the Health Education Program (HEP) arm, in the total sample, sex-, and MCI-subtype stratified analyses. **a** Changes in log-transformed plasma CRP levels across baseline, 3-month, and 9-month time-points in the Mindful Awareness Practice (MAP) arm, compared to the Health Education Program (HEP) arm, in the total sample. **b** Changes in log-transformed plasma CRP levels in female sub-group across baseline, 3-month, and 9-month time-points in the Mindful Awareness Practice (MAP) arm, compared to the Health Education Program (HEP) arm, according to sex. **c** Changes in log-transformed plasma CRP levels in the aMCI sub-group across baseline, 3-month, and 9-month time-points in the Mindful Awareness Practice (MAP) arm, compared to Health Education Program (HEP) arm, according to MCI subtype. **d** Changes in log-transformed salivary IL-1β levels in the male sub-group across baseline, 3-month, and 9-month time-points in the Mindful Awareness Practice (MAP) arm, compared to the Health Education Program (HEP) arm, according to sex. **e** Changes in log-transformed salivary IL-6 levels in male sub-group across baseline, 3-month, and 9-month time-points in the Mindful Awareness Practice (MAP) arm, compared to the Health Education Program (HEP) arm, according to sex.

A significant difference in plasma high-sensitivity c-reactive protein (hs-CRP) levels between MAP and HEP arms was observed at 9-month (*β* = –0.307, 95% CI = –0.559 to –0.054, *P* = 0.018), with MAP arm having significantly lower CRP level compared to HEP arm (Fig. 2a, Table 2, and Supplementary Fig. 1), after controlling for baseline covariates. Baseline CRP level (*β* = 0.824, 95% CI = 0.677 to 0.972, *P* < 0.001) was a significant covariate for the model. All the other covariates, including cardiovascular (CVS), metabolic, and inflammation-associated morbidities, were not included in the final model due to non-significance.

**Table 2:** Adjusted models for biomarkers, total sample.

Biomarkers	Time-points	Intervention, log-transformed adjusted mean (SE, 95% CI)	Control, log-transformed adjusted mean (SE, 95% CI)	Estimate (SE)	95% CI	*P-*value
Hs-CRP	Baseline	0.210 (0.063, 0.084 to 0.336)	0.217 (0.063, 0.091 to 0.343)	Reference	Reference	Reference
	3-month	0.181 (0.064, 0.053 to 0.309)	0.197 (0.065, 0.068 to 0.325)	−0.008	−0.210 to 0.193	0.933
	9-month	0.014 (0.084, −0.153 to 0.180)	0.327 (0.067, 0.194 to 0.461)	−0.307	−0.559 to −0.054	0.018*
IL-1β	Baseline	1.652 (0.101, 1.453 to 1.851)	1.619 (0.090, 1.440 to 1.798)	Reference	Reference	Reference
	3-month	1.767 (0.104, 1.561 to 1.973)	1.889 (0.098, 1.695 to 2.082)	−0.155	−0.479 to 0.170	0.346
	9-month	1.749 (0.117, 1.518 to 1.979)	1.799 (0.100, 1.602 to 1.996)	−0.083	-0.458 to 0.292	0.661
IL-6	Baseline	0.344 (0.108, 0.130 to 0.558)	0.392 (0.103, 0.189 to 0.595)	Reference	Reference	Reference
	3-month	0.459 (0.108, 0.245 to 0.673)	0.407 (0.114, 0.180 to 0.633)	0.100	−0.300 to 0.500	0.621
	9-month	0.506 (0.137, 0.234 to 0.778)	0.719 (0.116, 0.489 to 0.948)	−0.165	-0.605 to 0.276	0.462
BDNF	Baseline	7.238 (0.165, 6.910 to 7.565)	7.311 (0.174, 6.967 to 7.656)	Reference	Reference	Reference
	3-month	6.495 (0.168, 6.161 to 6.829)	6.455 (0.177, 6.104 to 6.806)	0.114	−0.557 to 0.784	0.736
	9-month	6.323 (0.243, 5.841 to 6.805)	6.628 (0.194, 6.243 to 7.013)	−0.231	−0.977 to 0.514	0.539
Cortisol	Baseline	−1.009 (0.062, −1.107 to −0.911)	−0.996 (0.050, −1.095 to −0.897)	Reference	Reference	Reference
	3-month	−1.001 (0.050, −1.099 to −0.903)	−0.873 (0.055, −0.982to −0.763)	−0.116	−0.303 to 0.072	0.223
	9-month	−0.892 (0.050, −1.014 to −0.769)	−0.844 (0.056, −0.955 to −0.733)	−0.035	−0.242 to 0.173	0.741
DHEA-S	Baseline	2.443 (0.028, 2.388 to 2.498)	2.456 (0.029, 2.400 to 2.513)	Reference	Reference	Reference
	3-month	2.441 (0.028, 2.386 to 2.497)	2.481 (0.029, 2.424 to 2.538)	−0.026	−0.115 to 0.063	0.561
	9-month	2.475 (0.035, 2.405 to 2.545)	2.476 (0.030, 2.417 to 2.536)	0.012	−0.096 to 0.120	0.827

Covariates controlled for in the linear-mixed model included the baseline values of the respective outcome variable, age, sex, years of formal education, time-points of the intervention, treatment arm, time-points, and treatment arm interaction term.

*Hs-CRP* high-sensitivity-c-reactive protein, *IL* interleukin, *BDNF* brain-derived neurotrophic factor, *DHEA-S* dehydroepiandrosterone sulfate.

*indicates *P*-value < 0.05.

For the total sample, there were no significant differences in IL-1β and IL-6 levels (Table 2 and Supplementary Fig. 1). No significant differences in plasma BDNF, salivary cortisol, DHEA-S levels were observed across all the three time-points in MAP when compared to HEP (Table 2 and Supplementary Fig. 1).

Exploratory sub-group analyses by sex showed that the effect of significantly improved hs-CRP at 9-month was only observed in females (*β* = –0.445, 95% CI = –0.700 to –0.189, *P* = 0.001) (Fig. 2b and Table 3a). The exploratory sub-group analyses of MCI subtypes showed that hs-CRP was significantly decreased only in the aMCI subtype (*β* = –0.569, 95% CI = –1.000 to –0.133, *P* = 0.012) and not the naMCI subtype (Fig. 2c and Table 3b). Furthermore, although whole-sample analyses did not yield significance, males had significantly decreased IL-1β (*β* = –0.607, 95% CI = –1.116 to –0.100, *P* = 0.021) (Fig. 2d and Table 3a) and IL-6 (*β* = –1.001, 95% CI = –1.761 to –0253, *P* = 0.011) (Fig. 2e and Table 3a) levels at 3-month and non-significant improvements at 9-month (*β* = –0.475, 95% CI = –1.000 to 0.052, *P* = 0.075) and (*β* = –0.637, 95% CI = –1.377 to 0.104, *P* = 0.090), respectively.

**Table 3a:** Adjusted models for biomarkers, sex-stratified sub-group analyses.

Biomarkers	Time-points	Intervention, log-transformed adjusted mean (SE, 95% CI)	Control, log-transformed adjusted mean (SE, 95% CI)	Estimate (SE)	95% CI	*P-*value
Male						
Hs-CRP	Baseline	0.173 (0.119, −0.069 to 0.415)	0.35 (0.153, 0.038 to 0.662)	Reference	Reference	Reference
	3-month	0.227 (0.119, −0.015 to 0.469)	0.143 (0.153, −0.169 to 0.456)	0.260	−0.222 to 0.743	0.272
	9-month	0.096 (0.194, −0.298 to 0.489)	0.318 (0.153, 0.005 to 0.63)	−0.045	−0.603 to 0.513	0.870
IL-1β	Baseline	1.861 (0.127, 1.601 to 2.12)	1.697 (0.128, 1.436 to 1.959)	Reference	Reference	Reference
	3-month	1.788 (0.136, 1.509 to 2.066)	2.232 (0.153, 1.92 to 2.543)	−0.607	−1.116 to −0.100	0.021*
	9-month	1.868 (0.154, 1.553 to 2.183)	2.179 (0.137, 1.899 to 2.459)	−0.475	−1.000 to 0.052	0.075
IL-6	Baseline	0.278 (0.158, −0.043 to 0.6)	0.259 (0.167, −0.082 to 0.6)	Reference	Reference	Reference
	3-month	−0.064 (0.158, −0.385 to 0.258)	0.924 (0.216, 0.483 to 1.364)	−1.001	−1.761 to −0.253	0.011*
	9-month	0.393 (0.237, −0.09 to 0.876)	1.01 (0.185, 0.634 to 1.386)	−0.637	−1.377 to 0.104	0.090
BDNF	Baseline	7.134 (0.219, 6.685 to 7.584)	7.339 (0.308, 6.708 to 7.97)	Reference	Reference	Reference
	3-month	6.343 (0.219, 5.894 to 6.792)	6.268 (0.308, 5.638 to 6.899)	0.279	−0.968 to 1.527	0.641
	9-month	6.554 (0.457, 5.619 to 7.49)	6.022 (0.358, 5.289 to 6.755)	0.737	−0.675 to 2.149	0.294
Cortisol	Baseline	−0.747 (0.066, −0.882 to −0.611)	−0.772 (0.077, −0.93 to −0.614)	Reference	Reference	Reference
	3-month	−0.792 (0.066, −0.928 to −0.657)	−0.882 (0.093, −1.07 to −0.693)	0.064	−0.204 to 0.332	0.621
	9-month	−0.732 (0.092, −0.919 to −0.545)	−0.772 (0.084, −0.944 to −0.6)	0.015	−0.290 to 0.320	0.921
DHEA-S	Baseline	3.013 (0.033, 2.946 to 3.079)	3.004 (0.046, 2.909 to 3.099)	Reference	Reference	Reference
	3-month	3.05 (0.033, 2.983 to 3.116)	3.038 (0.046, 2.944 to 3.133)	0.002	−0.137 to 0.142	0.968
	9-month	3.062 (0.052, 2.956 to 3.169)	3.122 (0.046, 3.027 to 3.216)	−0.068	−0.243 to 0.107	0.436
Female						
Hs-CRP	Baseline	0.192 (0.072, 0.049 to 0.336)	0.16 (0.065, 0.031 to 0.29)	Reference	Reference	Reference
	3-month	0.106 (0.073, −0.04 to 0.253)	0.2 (0.066, 0.068 to 0.333)	−0.126	−0.327 to 0.075	0.214
	9-month	−0.094 (0.085, −0.263 to 0.075)	0.318 (0.07, 0.179 to 0.458)	−0.445	−0.700 to −0.189	0.001**
IL-1β	Baseline	1.575 (0.12, 1.338 to 1.813)	1.584 (0.103, 1.379 to 1.789)	Reference	Reference	Reference
	3-month	1.754 (0.12, 1.516 to 1.992)	1.785 (0.109, 1.57 to 2.001)	−0.023	−0.417 to 0.370	0.906
	9-month	1.708 (0.138, 1.434 to 1.982)	1.679 (0.115, 1.451 to 1.907)	0.038	−0.425 to 0.501	0.872
IL-6	Baseline	0.306 (0.122, 0.063 to 0.549)	0.47 (0.112, 0.248 to 0.693)	Reference	Reference	Reference
	3-month	0.608 (0.122, 0.366 to 0.851)	0.344 (0.122, 0.102 to 0.586)	0.428	−0.015 to 0.873	0.058
	9-month	0.518 (0.144, 0.231 to 0.804)	0.681 (0.13, 0.424 to 0.938)	0.001	−0.497 to 0.498	0.998
BDNF	Baseline	7.376 (0.213, 6.952 to 7.8)	7.441 (0.198, 7.047 to 7.836)	Reference	Reference	Reference
	3-month	6.658 (0.222, 6.216 to 7.101)	6.637 (0.208, 6.223 to 7.051)	0.087	−0.749 to 0.923	0.835
	9-month	6.424 (0.262, 5.903 to 6.945)	6.933 (0.22, 6.494 to 7.371)	−0.443	−1.317 to 0.431	0.316
Cortisol	Baseline	−1.132 (0.06, −1.252 to −1.013)	−1.076 (0.058, −1.191 to −0.962)	Reference	Reference	Reference
	3-month	−1.1 (0.06, −1.219 to −0.98)	−0.897 (0.062, −1.021 to −0.774)	−0.146	−0.378 to 0.085	0.211
	9-month	−0.975 (0.073, −1.119 to −0.831)	−0.871 (0.066, −1.003 to −0.74)	−0.048	−0.300 to 0.205	0.709
DHEA-S	Baseline	2.206 (0.035, 2.137 to 2.276)	2.218 (0.032, 2.155 to 2.281)	Reference	Reference	Reference
	3-month	2.181 (0.035, 2.112 to 2.251)	2.24 (0.032, 2.177 to 2.303)	−0.047	−0.158 to 0.063	0.398
	9-month	2.227 (0.04, 2.148 to 2.306)	2.209 (0.034, 2.141 to 2.277)	0.030	−0.103 to 0.162	0.658

Covariates controlled for in the linear-mixed model included the baseline values of the respective outcome variable, age, sex, years of formal education, time-points of the intervention, treatment arm, time-points, and treatment arm interaction term.

*Hs-CRP* high-sensitivity-c-reactive protein, *IL* interleukin, *BDNF* brain-derived neurotrophic factor, *DHEA-S* dehydroepiandrosterone sulfate.

*indicates *P*-value < 0.05; **indicates *P*-value < 0.01.

**Table 3b:** Adjusted models for biomarkers, MCI subtype-stratified sub-group analyses.

Biomarkers	Time-points	Intervention, log-transformed adjusted mean (SE, 95% CI)	Control, log-transformed adjusted mean (SE, 95% CI)	Estimate (SE)	95% CI	*P-*value
Amnestic-MCI						
Hs-CRP	Baseline	0.231 (0.086, 0.058 to 0.405)	0.283 (0.109, 0.063 to 0.504)	Reference	Reference	Reference
	3-month	0.148 (0.092, −0.037 to 0.334)	0.268 (0.117, 0.032 to 0.503)	−0.068	−0.505 to 0.369	0.752
	9-month	−0.28 (0.131, −0.544 to -0.016)	0.34 (0.117, 0.104 to 0.577)	−0.569	−1.000 to −0.133	0.012*
IL-1β	Baseline	1.786 (0.158, 1.465 to 2.106)	1.625 (0.16, 1.3 to 1.95)	Reference	Reference	Reference
	3-month	2.088 (0.163, 1.758 to 2.419)	2.046 (0.166, 1.709 to 2.382)	−0.118	−0.652 to 0.416	0.654
	9-month	2.099 (0.2, 1.695 to 2.503)	2.027 (0.177, 1.67 to 2.384)	−0.089	−0.720 to 0.543	0.777
IL-6	Baseline	0.378 (0.119, 0.137 to 0.618)	0.46 (0.142, 0.173 to 0.747)	Reference	Reference	Reference
	3-month	0.62 (0.119, 0.38 to 0.86)	0.474 (0.148, 0.176 to 0.772)	0.228	−0.377 to 0.833	0.875
	9-month	0.864 (0.178, 0.506 to 1.222)	0.902 (0.16, 0.58 to 1.223)	0.045	−0.529 to 0.619	0.446
BDNF	Baseline	7.022 (0.243, 6.533 to 7.512)	7.203 (0.304, 6.59 to 7.816)	Reference	Reference	Reference
	3-month	6.375 (0.243, 5.886 to 6.864)	5.875 (0.304, 5.263 to 6.488)	0.680	−0.499 to 1.860	0.246
	9-month	6.097 (0.424, 5.243 to 6.951)	6.822 (0.326, 6.165 to 7.479)	−0.544	−1.851 to 0.763	0.407
Cortisol	Baseline	−0.790 (0.052, −0.894 to −0.686)	−0.748 (0.063, −0.874 to −0.622)	Reference	Reference	Reference
	3-month	−0.840 (0.052,−0.944 to −0.736)	−0.784 (0.065, −0.915 to −0.654)	−0.013	−0.235 to 0.208	0.902
	9-month	−0.688 (0.078,−0.846 to −0.531)	−0.597 (0.07, −0.737 to −0.457)	−0.049	−0.307 to 0.208	0.701
DHEA-S	Baseline	2.592 (0.032, 2.528 to 2.655)	2.611 (0.044, 2.523 to 2.7)	Reference	Reference	Reference
	3-month	2.556 (0.032, 2.493 to 2.62)	2.61 (0.044, 2.521 to 2.698)	−0.034	−0.191 to 0.123	0.657
	9-month	2.552 (0.048, 2.455 to 2.649)	2.575 (0.047, 2.48 to 2.669)	−0.003	−0.170 to 0.163	0.969
Non-amnestic-MCI						
Hs-CRP	Baseline	0.152 (0.098, −0.047 to 0.35)	0.158 (0.081, −0.005 to 0.321)	Reference	Reference	Reference
	3-month	0.182 (0.098, −0.017 to 0.38)	0.142 (0.081, −0.02 to 0.305)	0.045	−0.155 to 0.245	0.651
	9-month	0.089 (0.11, −0.132 to 0.309)	0.298 (0.084, 0.129 to 0.467)	−0.203	−0.459 to 0.053	0.118
IL-1β	Baseline	1.597 (0.149, 1.3 to 1.893)	1.554 (0.123, 1.308 to 1.799)	Reference	Reference	Reference
	3-month	1.606 (0.149, 1.309 to 1.902)	1.758 (0.132, 1.495 to 2.021)	−0.196	−0.600 to 0.209	0.337
	9-month	1.605 (0.155, 1.296 to 1.914)	1.652 (0.129, 1.395 to 1.909)	−0.091	−0.540 to 0.359	0.689
IL-6	Baseline	0.242 (0.191, −0.139 to 0.623)	0.264 (0.159, −0.053 to 0.58)	Reference	Reference	Reference
	3-month	0.248 (0.191, −0.132 to 0.629)	0.27 (0.174, −0.077 to 0.617)	0	−0.548 to 0.547	1.000
	9-month	0.291 (0.203, −0.113 to 0.695)	0.556 (0.168, 0.222 to 0.891)	−0.244	−0.832 to 0.344	0.411
BDNF	Baseline	7.821 (0.307, 7.2 to 8.442)	7.828 (0.271, 7.282 to 8.375)	Reference	Reference	Reference
	3-month	6.938 (0.314, 6.305 to 7.57)	7.243 (0.264, 6.708 to 7.778)	−0.298	−1.027 to 0.431	0.412
	9-month	6.844 (0.338, 6.164 to 7.523)	6.962 (0.3, 6.358 to 7.565)	−0.111	−0.892 to 0.670	0.778
Cortisol	Baseline	−1.217 (0.086, −1.388 to −1.045)	−1.163 (0.074, −1.31 to −1.015)	Reference	Reference	Reference
	3-month	−1.164 (0.086, −1.335 to −0.993)	−0.973 (0.083, −1.137 to −0.808)	−0.137	−0.403 to 0.129	0.305
	9-month	−1.085 (0.093, −1.27 to −0.9)	−1.019 (0.08, −1.179 to −0.859)	−0.011	−0.289 to 0.265	0.933
DHEA-S	Baseline	2.335 (0.049, 2.235 to 2.434)	2.347 (0.043, 2.26 to 2.435)	Reference	Reference	Reference
	3-month	2.371 (0.049, 2.271 to 2.47)	2.383 (0.043, 2.296 to 2.47)	0	−0.113 to 0.114	0.996
	9-month	2.406 (0.053, 2.299 to 2.512)	2.395 (0.043, 2.308 to 2.483)	0.023	−0.110 to 0.156	0.733

Covariates controlled for in the linear-mixed model included the baseline values of the respective outcome variable, age, sex, years of formal education, time-points of the intervention, treatment arm, time-points, and treatment arm interaction term.

*Hs-CRP* high-sensitivity-c-reactive protein, IL interleukin, *BDNF* brain-derived neurotrophic factor, *DHEA-S* dehydroepiandrosterone sulfate.

*indicates *P*-value < 0.05.

## Discussions

MAP improved inflammatory biomarkers in sex- and MCI subtype-specific manners. Overall, MAP improved hs-CRP levels, compared to the HEP. Interestingly, exploratory sub-group analyses by sex showed that the effect of significantly improved hs-CRP was mainly driven by the improvement observed in females. Furthermore, sub-group analyses by MCI subtypes showed that hs-CRP was improved only in the aMCI subtype, not the naMCI subtype. Although whole-sample analyses did not reach statistical significance, males had significantly improved IL-6 and IL-1β levels at 3-month and non-significant improvements at 9-month.

Our finding suggests that CRP is a modifiable risk factor of dementia that responded to mindfulness intervention in specifically older adults with MCI, consistent with prior findings shown in other sample populations^67,68^. Our finding extended the literature by suggesting that mindfulness might potentially delay cognitive decline by ameliorating CRP^67^. There are several pathophysiological mechanisms on how chronically elevated levels of CRP have been proposed to increase the risk of developing all-cause dementia^24–29^. Conversely, lower levels of CRP could be beneficial to older adults with MCI. One prior study has suggested that CRP is directly involved in the pathogenesis of atherogenesis and ischemic cerebrovascular diseases, contributing to the development of pathologies in the vasculature, a hallmark of vascular dementia (VaD)^29,69^. Furthermore, CRP has also been shown to act independently of systemic inflammation by crossing the blood-brain-barrier and directly effects neuro-inflammatory response in the brain^68,70^. Third, CRP has also been shown to co-localize with and further upregulate the two hallmarks of AD, amyloid-beta (Aβ) and phospho-tau proteins, in the brains of patients with AD^71,72^. Further study substantiated this hypothesis by showing that the interaction between CRP and Aβ1–42 heightened vascular abnormalities, such as enlarged lacunar counts and perivascular spaces^73^. Interestingly, when we performed exploratory sub-group analyses by sex, only females showed significant decreased hs-CRP levels upon completing the MAP. Our finding is consistent with prior literature, which found a significant relationship between CRP and cognitive decline in females only^25,26^. Our sample size comprises mainly female, the insignificance detected in male sub-group could either be attributed to males being not responsive to the intervention or statistically being underpowered. When the sample was stratified by MCI subtypes, statistical significance was only detected in the aMCI subtype, which is intriguing as this MCI subtype is a prodrome of Alzheimer’s type dementia^74^. On the other hand, increased CRP has been shown to precede both Alzheimer’s and vascular dementia^27^. Our results suggest that mindfulness may specifically ameliorate CRP in aMCI, but not naMCI, which warrant further studies in furthering the understanding of the underlying mechanism.

The overall analyses showed that IL-6 and IL-1β levels were not significantly improved at both 3-month and 9-month following MAP. One plausible reason being salivary cytokines might not be representative of blood cytokine levels. However, upon performing sub-group analyses, we found reduced IL-6 and IL-1β only in males, contrary to the finding of hs-CRP, suggesting the beneficial effect of MAP on systemic inflammation was present only in males. Interestingly, IL-6 is a well-known regulator of CRP production. For females, MAP could not have modulated CRP production through regulating IL-6 levels, as only CRP, but not IL-6 levels were significantly improved. Apart from IL-6, there are other biomarkers that had been shown to regulate CRP levels, including IL-1, IL-17, and tumor growth factor (TGF)-β^75,76^. Hence, it would be interesting to investigate these biomarkers comprehensively in future investigations, considering the sex effect. One plausible interpretation for improved cytokine levels in only males could be the underlying biological difference between the sexes; Among the candidates are sex hormones, as they could influence cognition, as well as inflammation^77,78^. Another study has also shown a sex-specific effect, showing that females benefited more from mindfulness intervention on emotional regulation^79^. Hence, taken together with the finding from hs-CRP, future studies should consider both sex and MCI subtype a priori when formulating hypotheses, as they affect both biomarkers and cognitive domains differentially and we have provided preliminary evidence that mindfulness intervention with MCI participants with different sexes and MCI subtypes might have differential effects on biomarkers.

We showed pilot empirical evidence in the MCI population on neuronal plasticity modulated by neurotrophic factors, and HPA-axis markers, represented by cortisol and DHEA-S. In contrary to the hypotheses and proposed theoretical mechanisms in the literature^17^, MAP did not increase BDNF and decrease stress-related biomarkers. There are two plausible interpretations; The limited sample size might have rendered the inability to detect significant changes in these biomarkers. Conversely, MAP might not have targeted these mechanisms in the sample MCI population. There have been conflicting findings on the effects of mindfulness on stress-related biomarkers in different sample populations. One proposition is that mindfulness may modulate cortisol and DHEA-S levels by altering the sensitivity of glucocorticoid receptor (GR) instead^80^. Regardless, these findings warrant replication in larger RCTs.

On the RCT implementation aspect, there were several issues on the feasibility and acceptability of the RCT worth discussing. First, recruitment strategies needed to cater to the characteristics of this population with cognitive impairment. The recruitment rate for this RCT is not high, about 43.65% (55 recruited out of 126 screened), lower than another RCT with MCI, which was 66.81%^81^. The 3-month average attendance and retention rates were approximately 88 and 89%, similar to other psychosocial interventions conducted with MCI participants, which were approximately 90 and 85%^14,82^. The instructors played a critical role in both the MAP and HEP. One takeaway lesson was that more patience and instructors’ interaction were needed to effectively engage this population, compared to cognitively intact older adults. Enhanced interaction with the instructors might explain the high attendance rate. For the homework and personal diary, the participants needed constant repetitions of instructions during the intervention sessions and reminders to practice at home, and to bring their personal diaries back to the research center for fidelity check purpose. Based on our interaction with the participants, we suspect that due to having MCI, many participants did not perform one or more of the aforementioned tasks. For future studies, we thus propose that a “cheat-sheet” containing a brief summary of the practices taught during each session to be distributed to the participants. Additionally, caregivers or family members could be approached during the screening and recruitment stage, to obtain consent and to be tasked to remind the participants constantly to practice the techniques and to keep track of the daily practices on behalf of the participants. Incorporating these implementation issues in future study designs may enhance engagement with participants with MCI. Lastly, no adverse event related to either the MAP or HEP intervention was reported, which suggests the feasibility and safety of the interventions.

We noted several limitations that warrant discussion. Despite the encouraging findings, due to the small sample size, they are preliminary and thus requiring validation in larger RCTs, specifically on the results of exploratory sub-group analyses. With a larger sample size, more diverse variables could be included a priori to test for their potential modifying effects, in this instance, sex and MCI subtypes. Second, as an intervention of psychosocial nature, inadvertently there could be residual confounding effects when limited number of covariates were examined and controlled for. Therefore, in future studies of mindfulness intervention, researchers should collect variables pertinent to both MCI and biomarkers, in particular exercise, diets, intakes of supplements, changes in medication consumptions. Additionally, blinding of the participants to the assigned interventions was not feasible. This issue has also been discussed previously^83^, with one potential mitigation to be employed in future studies would be to use sham meditation as the active control arm^18^. On the other hand, infeasibility in blinding also reflected a more realistic and naturalistic setting in examining the effects of mindfulness intervention. Another limitation was that we were unable to determine the adherence rate of the participants to the interventions. Likely due to cognitive impairments, most of the participants either did not practice at home, forgot to keep track of their practices at home, or forgot to return their diaries, causing issues in conducting fidelity check. This issue warrants heightened attention in conducting adherence and fidelity check in future studies involving participants with MCI. Lastly, due to the limitations in logistics and human resources, the time interval for saliva collection was relatively long and only a single sample of saliva was collected for each participant at each time-point. Thus, the salivary cortisol levels reported here should be interpreted with caution. For higher accuracy and reliability, the ideal saliva sampling method would be to collect saliva samples at multiple time-points throughout the day and taking the reading from the area under the curve or the concentration versus time curve with respect to zero^84–86^. Furthermore, there is contention within the literature on how well salivary markers reflect peripheral or brain-based biomarkers in general. Compared to salivary cytokines, which have less evidence supporting correlations to their corresponding blood marker levels, salivary cortisol has been demonstrated to be highly correlated with unbound cortisol in the blood^87^.

Despite these limitations, this study represents a significant advancement in the field in several aspects. First, we employed an RCT design, with the active control arm controlled for several intervention components, which were not specific to mindfulness, for instance, an equal amount of instructor’s attention^18^, the time of the day and week^88^, and the length of the sessions^88^. This study design minimized residual confounding effects, by ensuring that the two study arms differed mainly in the interventions being compared;^89^ Second, we concomitantly examined a range of biomarkers representing different biological mechanisms, which enabled us to narrow down the specific effects of MAP in MCI. Third, we have also addressed one of the main limitations present in the literature, namely the short follow-up period, by adding booster sessions from 3- to 9-month. In the literature, the average follow-up period of most mindfulness interventions was a relatively short eight weeks. To our knowledge, this study is one of the longest follow-up RCTs of mindfulness intervention. With this extended follow-up period, we elucidated the long-term effects of mindfulness, therein extending the literature.

In all, we demonstrated proof-of-concept of mindfulness intervention in ameliorating biomarker perturbations implicated in cognitive decline and dementia in older adults with MCI. These preliminary findings are encouraging, coupled with the fact that mindfulness is a low-cost and self-directed intervention, in which the older adults can practice without time and space constraints. Owing to the pilot nature and small sample size of the study, there is still limited evidence at this stage to recommend mindfulness intervention to older adults with MCI in clinical practice. Further validation, particularly in large-scale RCT, is warranted. Some important future directions identified in this study include a priori examinations of the effects of sex and MCI subtypes to delineate their modifying effects on the outcomes of mindfulness interventions with older adults with MCI. Lastly, neurocognitive data are necessary to examine if mindfulness could improve cognitive functions. Future studies should examine how pervasive mindfulness could ameliorate declines in different cognitive domains affected in older adults with MCI, taking into account sex and MCI subtypes.

## Supplementary information

Supplementary text_MAP-detailed explanations of each practice

Supplementary Figure
