# Supplementary material for: Mindfulness improves inflammatory biomarker levels in older adults with mild cognitive impairment: a randomized controlled trial
Source: Transl Psychiatry. 2020 Jan 21;10:21. doi: 10.1038/s41398-020-0696-y (PMC7026149; doi:10.1038/s41398-020-0696-y)
Supplement: Supplementary file 1 — Supplementary text_MAP-detailed explanations of each practice [file 41398_2020_696_MOESM1_ESM.docx]

# Supplementary text

# MAP-detailed explanations of each practice

# Mindfulness of the senses practice involved teaching the participants paying attention to their senses while performing daily activities, such as brushing teeth. During the practice of mindful breathing and body scan practice, the participants were guided by the instructor to place their attention on one part of the body at a time, focusing from the toes to the head or vice versa, while directing their breath to the respective areas of the body and relaxing the muscles at the area. Movement nature meant practice involved movement based on feldenkrais method to restore natural coordination and movement mobility, with two of the cornerstones of feldenkrais method involves “Awareness Through Movement”® and breathing awareness [1]. For the visual-motor coordination task, the participants were guided to perform a variety of cognitively demanding tasks that required the fine coordination of the visual and motor systems, such as using one hand to perform a task using rubber bands and then switching quickly to performing the same task using the other hand, back and forth to be repeated a few times. Lastly, mindful stretching aimed to relax the muscles of the participants, by requiring them to stand up and to stretch their muscles mindfully, while sighing and releasing the tensions felt in the muscles.

**Reference**

1. Alon, R., *Mindful spontaneity: lessons in the Feldenkrais method*. 1996: North Atlantic Books.
