# Supplementary material for: Mindfulness improves inflammatory biomarker levels in older adults with mild cognitive impairment: a randomized controlled trial
Source: Transl Psychiatry. 2020 Jan 21;10:21. doi: 10.1038/s41398-020-0696-y (PMC7026149; doi:10.1038/s41398-020-0696-y)
Supplement: Supplementary file 2 — Supplementary Figure [file 41398_2020_696_MOESM2_ESM.docx]

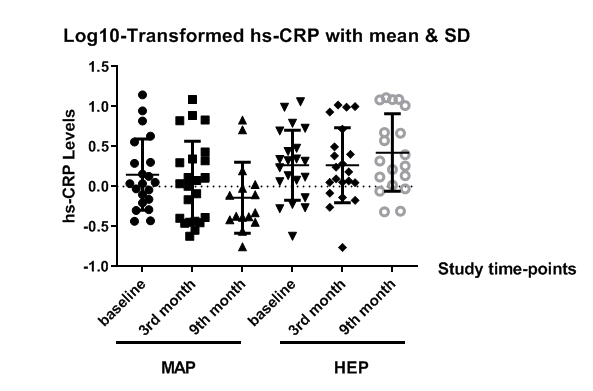

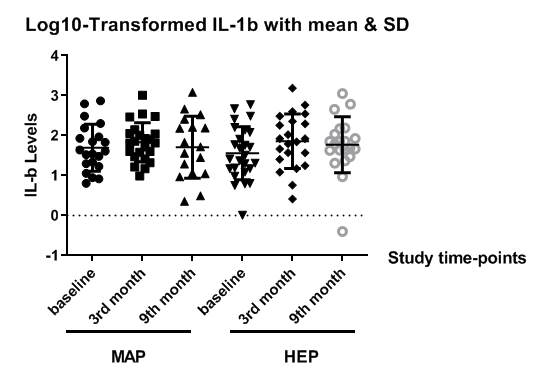

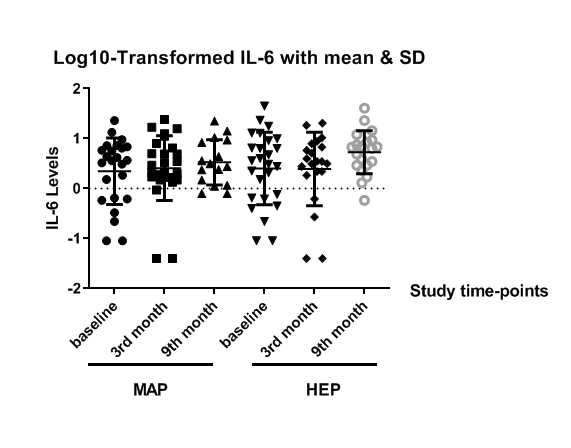


*P*=0.018

**(a)**

**(b)**

**(c)**

**(c)**


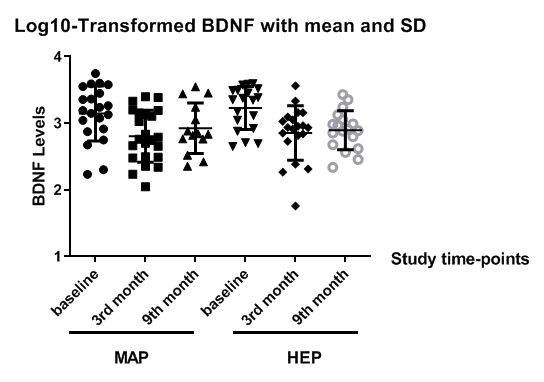


**(d)**


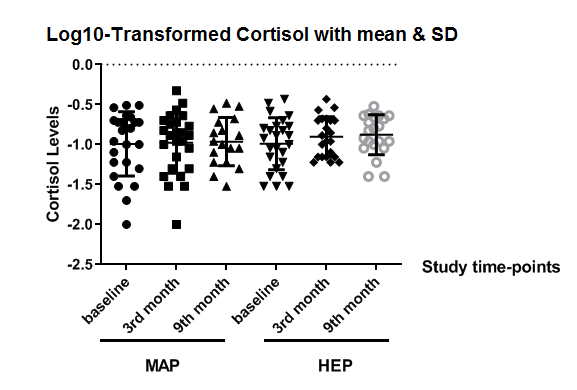


**(e)**


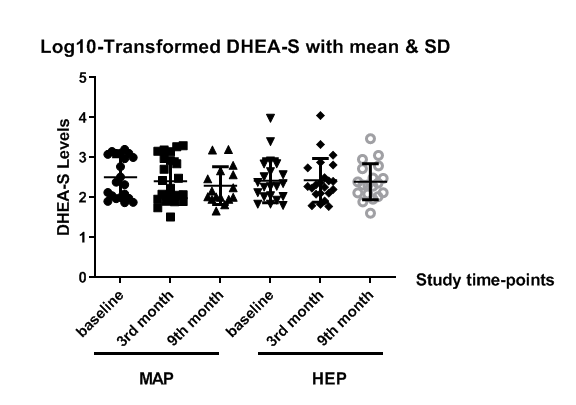


**(f)**

**Supplementary Figure. Dot plots depicting the changes of biomarker levels in the interventional arm, Mindful Awareness Program (MAP), and the active control group, Health Education Program (HEP). (a): high-sensitivity C-reactive protein (hs-CRP), (b): Interleukin (IL)-1β; (c) IL-6; (d): BDNF (brain-derived neurotrophic factor); (e): Cortisol; (f): DHEA-S (dehydroepiandrosterone).**
